# Non-booking for antenatal care and risks for vertical HIV transmission among women in Chitungwiza, Zimbabwe: a cross-sectional study

**DOI:** 10.1186/s12884-022-05131-x

**Published:** 2022-11-05

**Authors:** Patricia Mandima, Nikki Schaay, Bernard Ngara, Martina Lembani

**Affiliations:** 1 University of Zimbabwe- Clinical Trials Research Centre, 15 Philips Avenue Belgravia, Harare, Zimbabwe; 2 University of the Western Cape, School of Public Health, Robert Sobukwe Road, Bellville, Cape Town, 7535 South Africa; 3 Department of Primary Health Care Sciences, University of Zimbabwe College of Health Sciences, Mazowe Street, Parirenyatwa Complex, P. O Box A178 Avondale, Harare, Zimbabwe

**Keywords:** Booking, Pregnancy, HIV, PMTCT, Women

## Abstract

**Background:**

The success of prevention of mother to child transmission of HIV (PMTCT) programs dependents on pregnant women accessing antenatal care (ANC) services. Failure to access ANC throughout the course of pregnancy presents a missed opportunity to fully utilize PMTCT services and a high risk for vertical HIV transmission. Whilst not booking for ANC was about 6% in Zimbabwe, according to the 2015 Zimbabwe Demographic and Health Survey, it is important to determine the local burden of pregnant women both un-booked for ANC and living with HIV. in Chitungwiza city, to inform local response. This study aimed at determining the proportion of women un-booked for antenatal care and among them, the proportion of women who were with HIV and to identify risk factors associated with not-booking for ANC in Chitungwiza city in Zimbabwe.

**Methods:**

A cross-sectional study was conducted involving a review of clinic records for 4400 women who received postnatal care at all 4 maternity clinics in Chitungwiza city between 01 January 2017 and 31 December 2017. Bivariate and multiple logistic regression analysis with Chi squared test were used to determine risk factors associated with booking status while adjusting for other study variables. All statistics tests’ decisions were concluded at 5% level of significance. All data analysis was performed using STATA (version 13) statistical package.

**Results:**

A total of 4400 women were attended to and of these, 19% were un-booked for ANC, while a total of 3% of the women were both un-booked and living with HIV. The women with HIV were 0.24 times less likely to book for ANC than HIV negative women, adjusted OR = 0.76 (95% CI: 0.61–0.98). Women aged 20–34 years were 1.3 times more likely to book than the teenagers, adjusted OR = 1.3 (95% CI: 1.04–1.62).

**Conclusion:**

The proportion of women not booked for ANC of 19% was unexpectedly high. With 3% of pregnant women in Chitungwiza having both HIV and no access to ANC, the risk for vertical HIV transmission remains. More need to be done to improve ANC access, targeting teenage mothers and those living with HIV who are more less likely to access ANC.

## Background

At the end of 2020, approximately 38 million people were living with human immunodeficiency virus (HIV) and of these, 1 million were children aged 0 to 9 years [1]. In the same year, 1.5 million people became newly infected with HIV around the world and this included 160,000 children under the age of nine [1].

Approximately 1.3 million women living with HIV got pregnant in 2018 [2] and Mother- to- Child Transmission of HIV (MTCT) in-utero or through breastfeeding is responsible for over 90% of all paediatric HIV infections [3]. Prevention of Mother-to-Child Transmission of HIV (PMTCT) is therefore critical in the prevention of paediatric HIV infection. To prevent HIV infection occurring in children, the World Health organisation (WHO) recommends a range of strategies, namely: the prevention of HIV in women of childbearing age, the prevention of unwanted pregnancies, PMTCT with anti-retroviral therapy (ART) in HIV positive pregnant women and infant prophylaxis for HIV exposed infants [4]. Antenatal care (ANC) plays a vital role in one of these strategies, namely, PMTCT, since it is the entry point for pregnant women into health care services. Women, who for various reasons fail to utilise ANC, would unfortunately have missed the opportunity to fully utilize PMTCT services and are at high risk for maternal HIV transmission.

Antenatal care refers to the ongoing health services provided to women during pregnancy in the formal health system. It is meant to screen out pregnant women for potential obstetric complications, non-communicable and infectious diseases and provide therapeutic and preventive interventions to minimise maternal and fatal adverse pregnancy outcomes [5]. In 2002, the WHO issued a set of ANC guidelines for developing countries which incorporated what was proposed in relation to the prevention of mother to child transmission of HIV [5].

Henceforth, ANC became the focal point for access to PMTCT services by pregnant women [6]. Unfortunately, about 14% of pregnant women worldwide have no access to a single ANC visit with trained healthcare workers [7]. In Zimbabwe, the HIV prevalence among pregnant women is 16.7% and about 7% of all pregnant women in Zimbabwe fail to access ANC services throughout pregnancy for various reasons [8]. These women, un-booked for ANC, unfortunately miss the opportunity to fully utilise PMTCT services and those with HIV become very high risk for MTCT. Without intervention, the risk for MTCT is 15 to 45% [9] but can be as low as below 1% with PMTCT [10].

Many factors have been described which affect access to antenatal care. They include women’s perceptions on the importance of ANC and health problems during pregnancy. It has been suggested that marital status, parity, health worker – related factors, the costs of ANC and the fear of HIV testing were some of the factors influential in the utilisation of antenatal care services [11].

A systematic review by Simkhada (2008) further identified maternal education, husband’s education, availability of ANC services, household income, women’s employment status, media exposure and having a history of obstetric complications along with a women’s marital status, as factors affecting access to ANC. Cultural beliefs and ideas about pregnancy also influenced ANC utilization [12]. Many studies have shown that poor socio-economic status is associated with poor use of antenatal services [13–15]. For example, attending ANC is associated with costs even in facilities where the service is offered free. Such costs include those required for transport and the time spent at the ANC clinic.

Women who have completed secondary school or those with a tertiary education are noted, in many studies, as better utilisers of antenatal care services compared to those with no education and those who did not complete primary or secondary education [16–18]. Educated women may have a better perception of the importance of ANC, have a better understanding of health education messages and are less intimidated to approach health care workers [11]. Similarly, it has been noted that professional women are good users of antenatal care services compared to unemployed women [12, 17]. Women who get pregnant at ages less than 20 are usually not financially independent, unmarried and have not yet completed secondary or tertiary education and have been identified as poor utilisers of antenatal care services. In some settings, this can be worsened by poor pregnancy disclosure and social stigma [11, 16].

Researchers have noted that lower parity, a planned pregnancy, and past pregnancy complications were associated with better utilisation of ANC [16, 17].

.Interestingly, most studies focusing on the risk factors associated with poor utilisation of ANC tend to focus on the comparison between early bookers and late bookers and do not focus on the risk factors for not booking at all. Galvin (2001) showed that non-bookers were likely to be of lower socio-economic status compared to booked women in Harare, Zimbabwe. However, this study was done at a referral hospital where most of the non-bookers had been referred from rural areas which does not represent the urban Chitungwiza population [19].

Despite the fact that anti-retroviral drugs have been made available free of charge in the public health sector since 2002 and that HIV care is decentralized so that PMTCT services are available at all maternity health centres in Zimbabwe, some pregnant women appear to still find it difficult to access these services [8]. These women either present at maternity clinics as unbooked clients during labour or well after giving birth without having had a single contact with antenatal care services prior to the labour or delivery. Those newly diagnosed with HIV would then have missed taking ART during pregnancy and one of the obvious results of this is the children born are at high risk (15–45%) of contracting HIV [9], when such could have been prevented.

Whilst there is the Harare provincial data showing an un-booked proportion of 6% [8], the local burden of un-booked women in the city of Chitungwiza is not readly available. The proportion of these un-booked women who also have HIV, is again not readily available. Even though the data are collected and are present in maternity records, they are not available as aggregate data. A study by Chadwick et al., which recruited a population of unbooked postpartum women with HIV, indicated that the proportion of unbooked women in Chitungwiza could be way higher than the 6% estimated for Harare province where Chitungwiza is located [20].

This study therefore aimed at determining the proportion of women un-booked for antenatal care and among them, the proportion of women who were HIV positive, and to identify some of the risk factors for un-booking in the city of Chitungwiza. Determining the local burden of non-utilisation of ANC services is essential in identifying what local health promotion strategies and health service actions and resources are needed to bring such pregnant women into care. Determining the proportion of pregnant women both unbooked for ANC and living with HIV is important in assessing the risk of vertical HIV transmission in the city and what effort is still needed for the elimination of paediatric HIV.

## Methods

### Definition of key phrases

**Table:** 

**Booked women**	Refers to women who attended at least one antenatal care visit during a pregnancy.
**Unbooked women**	Refers to women who did not have contact with antenatal care services during a pregnancy and *only* present at a clinic during labour or after delivery.

### Study design

The study used an analytical cross-sectional design based on retrospective review of clinic records of women who delivered and/or received postnatal care at the 4 maternity clinics in Chitungwiza between 01 January 2017 and 31 December 2017. This design was chosen in order to determine at one point, frequencies and distribution of ANC booking status and outcome variables of age, HIV status, and the clinic attended. Since the study was a retrospective review, the number and types of outcome variables collected was limited to what was available in the maternity records.

### Description of study setting

The study was carried out in Chitungwiza which is the third most populous city in Zimbabwe, situated about 30 km south of the capital city, Harare. Chitungwiza is an urban city surrounded by urban Harare on the northern side, peri-urban informal settlements on the western side, then a rural setting on the south and eastern sides. It has a population of 356,840 and an annual growth rate of 1%. About 64% of the population is comprised of adolescents and adults over 15 years of age, among whom 30% are women of child-bearing age (15–49 years) (Unpublished Chitungwiza city health statistics). The overall adult prevalence of HIV in Chitungwiza in the 15–49-year age group is estimated to be 15%, with women harbouring higher burden of disease at 18% prevalence in comparison with 12% among men. The prevalence among antenatal clinic attendees is 16%, with Chitungwiza having high teenage pregnancy rate with 27% of teenagers having begun childbearing (unpublished data). The city is divided into 4 urban sub-districts, Seke North, Seke South, Zengeza and St Mary’s and each sub-district is serviced by a polyclinic with a similar name as the sub-district. The maternity side of the clinics is staffed by midwives who refer complicated cases to the government-run Chitungwiza General Hospital situated about 4–6 km from each clinic. The clinics have been offering PMTCT services since 2002 to the Chitungwiza urban and peri-urban community. There is also a private hospital in the city and many private doctors. The study was carried out in 2018 at the 4 polyclinics through collection of data from clinical records of all the women who delivered at the clinics from 01 January 2017 to 31 December 2017.

### Study population and sampling

The study population included all women who were registered to have received postnatal care at the 4 study maternity clinics between 01 January 2017 and 31 December 2017.

### Sample size determination

The Dobson’s sample size calculation was used to determine minimum sample size, using level of significance of 5% and the expected proportion (p) of booked women in the target population of 93% and margin of error of 5%. The total minimum number of participants required at 5% level of significance, assuming *p* = 93% [8] and 5% error of margin was 400. However, since the clinic records were already available, a total population sample size of 4400 was used in the analysis. Use of the largest available sample was done to improve the validity and reliability of the results by further reducing the impact of chance.

### Inclusion criteria

The study included data on women who had delivered at term at any one of the 4 study maternity clinics between 01 January and 31 December of 2017. Women who had delivered at term outside the healthcare system and presented to the clinics for postnatal care were included in the study. All term deliveries were included whether the babies were alive or not.

### Exclusion criteria

The study excluded women with miscarriages, abortions and preterm deliveries. ANC booking status for these women could not be ascertained as those unbooked still had time to book had the pregnancies reached term.

Women who were referred, during labour, for further care and did not deliver at the clinics were excluded from the study because they did not have complete data in the clinic records.

### Data collection

Data collection started after receiving approval from UWC IRB, MRCZ and from Chitungwiza City Ethics Committee. Upon receiving permission to perform the study at the clinics by the Chitungwiza City Health Department, the clinic managers of the 4 clinics were notified about the study and requested to give study staff access to maternity records. A nurse trained in Good Clinical Practice and with experience in clinical trials was contracted to abstract data for the study. Training on the study, the data extraction tools, and process was done prior to initiating the work. The first visit was done together with the study investigator for onsite training and to verify competence. The nurse visited each clinic once weekly from August to November 2018 to access the maternity records and to abstract data from the maternity registers to a data extraction sheet. Data on ANC booking status, age, HIV status, and clinic attended were collected. Names were not recorded during data abstract; numbering was used instead. The data extraction tool was reviewed regularly by the investigator for completeness. Spot checks were done by the investigator once monthly at each clinic to verify accuracy and any discrepancies between the source clinic data and the data extraction tool.

### Data management

The completed and reviewed data extraction tool was entered onto an excel spreadsheet by a data entry cleck. Data cleaning was repeated after entry onto the Excel spreadsheet by the investigator, comparing the data extraction tool and spreadsheet to ensure completeness and accuracy of the data. Data entry and quality check continued from February 2019 to April 2019.

### Data analysis

All the data on booking status, age, HIV status, and clinic attended were categorized. Firstly they were summarized using frequencies and percentages. Chi-square test was used in bivariate analysis to determine association between participants’ booking status and age, booking status and HIV status, and booking status and attended clinic. Associations showing *p* values of less than 0.05 were further analysed by multiple logistic regression to determine the association with booking status while adjusting for other study variables. All statistic tests decisions were concluded at 5% level of significance. All statistical analyses were performed in STATA software package version 13.

## Results

A total of 4400 women delivered and/or received postnatal care for term babies at the 4 maternity clinics in 2017. Of these, 820 (19%) were unbooked and 3580 (81%) were booked for ANC. Of the 3580 booked women, 352 (9,8%) had HIV infection whilst 118 (14.4%) of the 820 unbooked women had HIV. A total of 470 (11%) of all the women had HIV infection and 3% were both unbooked and had HIV. Out of the total, 597(14%) were aged below 20 years, 3304(75%) were aged 20–34 years and 499 (11%) were above 34 years old. The women were recruited from 4 clinics, 1397 (32%) were seen at Seke South Clinic, 1264 (28%) were from Seke North Clinic, 1012 (23%) were from St Mary’s clinic and 727 (17%) from Zengeza clinic. See Table 1.

**Table 1:** General characteristics of women who received postnatal care in Chitungwiza Clinics.. The variables are presented as N (%)

Variable	Frequency (percentage in study population)
Booking status	
Booked	3580 (81)
Unbooked	820 (19)
Age (years)	
< 20	597 (14)
20–34	3304 (75)
> 34	499 (11)
HIV Status	
HIV positive	470 (11)
HIV negative	3930 (89)
Clinic attended	
Seke North	1264 (28)
Seke South	1397 (32)
St Mary’s	1012 (23)
Zengeza	727 (17)

On risk factors, being attended at St Mary’s clinic, ages of below 20 and HIV positive status were significantly associated with not booking for ANC, as shown in Table 2.

**Table 2:** Multivariate logistic analysis of factors associated with booking

Variable	Booked ***N*** = 3580 (81)	Unbooked ***N*** = 820 (19)	aOR (CI)
Age (years)			
< 20	461 (77)	136 (23)	
20–34	2737 (83)	567 (17)	0.022 (1.04–1.62)
> 34	384 (77)	115 (23)	0.464 (0.83–1.51)
HIV Status			
HIV positive	352 (75)	118 (25)	0.037 (0.61–0.98)
HIV negative	3228 (82)	702 (18)	
Clinic attended			
Seke North	1046 (83)	218 (17)	
Seke South	1144 (83)	253 (17)	0.138 (0.69–1.05)
St Mary’s	776 (77)	236 (23)	0.02 (0.62–0.960
Zengeza	614 (84)	113 (16)	0.902 (0.76–1.27)

In multivariate analysis, HIV positive women were 0.24 times less likely to book than HIV negative women, Adjusted OR = 0.76, (95%CI: 0.61–0.98). Women attended at St Mary’s clinic were 23% less likely to book compared to Seke North women, Adjusted OR = 0.77 (95% CI: 0.62–0.96). Women aged 20–34 years were 1.3 times more likely to book than those aged < 20 years, Adjusted OR = 1.3 (95% CI:1.04–1.62). See Table 2.

## Discussion

The study identified the burden of unbooking for ANC in Chitungwiza to be 19%. This burden is much higher and even triples the 6% unbooking suggested for Harare province where Chitungwiza is located [8]. Percentages of women not utilizing ANC of greater than 19% have been reported for some Sub Saharan countries. A study involving 235,207 women who have had at least one birth within 5 years preceding Demographic and Health surveys of 31 Sub-Saharan Africa conducted between 2010 and 2018 showed that 13% of the women did not book for ANC. Countries with the highest percentage of women who did not book for ANC during pregnancy include Nigeria (26.1%) Ethiopia (34.8%) and Chad (41.8%). The lowest percentages of less than 1% were reported for Rwanda, Gambia and Burundi. Although Harare provincial data on the percentage of un-booked women f are available from the 2015 ZDHS, there was no available local level data for the burden of unbooking specifically for Chitungwiza. These data are important for local outreach and health service planning and the intention of this study was to contribute to providing such local-level data. It must be borne in mind that the ZDHS results are based on women responding to a question on their booking status on the last baby they had in the past 5 years. According to the ZDHS 2015, *“women who had a live birth in the 5 years preceding the survey were asked a number of questions about maternal and child health care. For the last live birth in that period, mothers were asked about antenatal care (ANC) during the pregnancy, assistance during delivery, the location of the delivery, and the timing of postnatal care.”* [8] In this case, the desire to please the interviewer and recall bias cannot be ruled out and could result in an underestimate of the problem of unbooking based on experiences with un-booked women in the city [20]. It was the intention of this study to provide more reliable local data on the burden of unbooking - based on a more reliable method i.e. using delivery records.

The available national data on ANC access is not linked to antenatal HIV prevalence data, hence national, provincial, and local data on the percentage of un-booked pregnant women with HIV infection is not readily available. In this study, 3% of all women delivering and/or receiving postnatal care at Chitungwiza maternity clinics were both un-booked and living with HIV. These are women who did not access PMTCT services offered during ANC and were at high risk for perinatal HIV transmission. Unless such women are identified and brought into care, children will continue to be born with HIV amidst tried and tested HIV prevention measures. Though there are data on HIV seroprevalence among unbooked women, to our knowledge, this is the first study to identify the percentage of pregnant women who were both unbooked and living with HIV in Zimbabwe.

The study also found that the ANC prevalence of HIV was 11% This percentage was lower than the Zimbabwe national 2015 ANC prevalence of 16% [8]. The lower percentage could be explained by the declining national HIV incidence which went down from 1% in 2015 to 0.54% in 2017 [21].

Unbooking was significantly higher in St Mary’s compared to the other 3 clinics. This could be explained by the lower socio-economic status of St Mary’s as it services more peri-urban overcrowded informal settlements compared to other clinics. It is common knowledge that peri-urban informal settlements are associated with poverty and unemployment which negatively affect utilisation of health services requiring user fees [13–18].

Women less than 20 years and those above 34 were less likely to book compared to the 20–34-year-olds and this was clinically significant. These results were in contrast with the 2015 ZDHS data which showed highest ANC attendance of 94.6% among those aged < 20 years compared to 93% in those above 20 years [8].

A major limitation of the study is that it was designed as a cross-sectional study and can therefore only capture association but not causation. The study was not able to capture all un-booked women from Chitungwiza as some women deliver at home under the care of traditional birth attendants and do not visit the antenatal clinics. As Chitungwiza is only 28 km from Harare (the capital of Zimbabwe), some women opt to seek ANC at Harare clinics and others in private centres in Chitungwiza, thus were not captured by the study and not represented in the study.

## Conclusion

This study aimed to provide data on burden of unbooking specifically for Chitungwiza city and so inform the planning of local interventions that will prioritise efforts to improve ANC utilisation in the city. The burden of unbooking was found to be 19% which is much higher than the expected 6%.

The burden of unbooked women who were also living HIV, who could have also missed opportunity for PMTCT during pregnancy was also determined to be 3%. Hence unbooking for ANC is still a contributing factor to the persistent perinatal HIV transmission.

Programs that target HIV positive women, pregnant teenagers and older pregnant women and women from peri-urban areas could help improve ANC attendance hence PMTCT uptake in Chitungwiza. Lessons can be learnt from Tanzania where the use of community health workers (CHWs) to identify pregnant women in the community and counselling on ANC attendance was very effective in improving ANC and PMTCT uptake among pregnant women [22].

## Data Availability

The data can be available upon request and approval by the corresponding author.

## Abbreviations

AIDS: Acquired Immunodeficiency Syndrome
ANC: Antenatal care
ART: Anti-retroviral therapy
AZT: Zidovudine
CI: Confidence Interval
eMTCT: Elimination of Mother-to-Child Transmission
HIV: Human Immunodeficiency virus
LIC: Low-income countries
MoHCC: Ministry of Health and Child Care of Zimbabwe
MRCZ: Medical Research Council of Zimbabwe
MTCT: Mother-to-child transmission
NAC: National AIDS Council
OR: Odds Ratio
PMTCT: Prevention of mother-to-child transmission of HIV
PrEP: Pre-exposure prophylaxis
sdNVP: Single dose nevirapine
STATA: A statistical analysis software
STI: Sexually transmitted infection
UNAIDS: Joint United Nations Programme on HIV and AIDS
UNICEF: United Nations International Children Emergency Fund
WHO: World Health Organization
UWC: University of Western Cape
ZIMSTAT: Zimbabwe National Statistical Agency
ZDHS: Zimbabwe Demographic and Health Survey
